# Does Emission Trading Boost Carbon Productivity? Evidence from China’s Pilot Emission Trading Scheme

**DOI:** 10.3390/ijerph17155522

**Published:** 2020-07-30

**Authors:** Di Zhou, Xiaoyu Liang, Ye Zhou, Kai Tang

**Affiliations:** 1 School of Mathematics and Statistics, Guangdong University of Foreign Studies, Guangzhou 510006, China; 201610098@oamail.gdufs.edu.cn; 2 School of Business, Guangdong University of Foreign Studies, Guangzhou 510006, China; XueYuLang19@163.com (X.L.); zy15972967500@163.com (Y.Z.); 3 School of Economics and Trade, Guangdong University of Foreign Studies, Guangzhou 510006, China

**Keywords:** emissions trading, carbon productivity, DDD, industrial heterogeneity, technological progress

## Abstract

As the country with the largest carbon emissions globally, the effective operation of China’s carbon emissions trading scheme (ETS) is of great importance to the global community in terms of mitigating climate change. This paper considers China’s pilot ETS launched in 2013 as a quasi-natural experiment. Exploring provincial industrial-level data that are more in line with the ETS coverage, the difference-in-difference-in-difference (DDD) model is used to evaluate the impact of the ETS on carbon productivity. Considering different pilot regions and industries, we also analyze the heterogeneous effect of ETS. Moreover, the mediating effects of technical progress and capital investment are explored. We find that China’s pilot ETS boosted carbon productivity. Among pilot regions, the best policy effectiveness appeared in Beijing, while the weakest effectiveness appeared in Chongqing. Among the pilot industries, the pilot ETS had better effectiveness in petrochemical and electric power industries and weaker effectiveness in building materials and transportation industries. Additionally, the pilot ETS promoted carbon productivity through both technological progress and capital investment, and the former contributed more. Our findings can provide empirical references and policy implications for nationwide implementation of ETS to further promote low-carbon economic transformation.

## 1. Introduction

The issue of global warming has aroused widespread concern around the world [1]. Changes in the global climate have significant impacts on the environment, economic activities, and residents’ health [2,3,4]. Excessive emissions of greenhouse gases caused by energy consumption, such as carbon dioxide (CO_2_), are believed to be the main cause of such a warming [5,6,7].

Accordingly, China, the world’s largest energy consumer and CO_2_ emitter, has promised to stop its increase in carbon emissions and reduce the emission intensity by 60% to 65% compared to the 2005 level before 2030 [8]. In the meanwhile, China faces a major challenge of achieving energy conservation and emission reduction without sacrificing economic development as a developing economy with a population of 1.4 billion [9,10,11]. To tackle this challenge, increasing carbon productivity has been identified as an effective way [12].

The proposed indicator relates to the carbon productivity defined as the amount GDP per unit of carbon emissions which focuses on describing the beneficial output of carbon emissions [13]. Increasing carbon productivity is key to addressing the twin challenges of mitigating climate change and managing economic growth. Therefore, it could be applied to evaluate the efforts to tackle climate change and the level of low-carbon economy in a region [13], and is also an important indicator to monitor green growth progress in industries [14]. Related studies have used it to assess how environmental regulation influences productivity [15] and the emissions performance of a region or multiple industries over time [16].

In order to realize its reduction commitments, China needs to further increase carbon productivity. To this end, the Chinese government officially introduced an emissions trading scheme (ETS) in 2011 [7]. Seven regions were initiated as carbon trading pilots. In 2013, those pilot regional carbon trading markets (CTMs) were officially established (Table 1).

Previous studies have found that the ETS can promote carbon mitigation [17,18] and reduce carbon intensity, which is defined as the ratio of carbon emissions to economic output [19,20]. Will China’s newly established ETS also promote carbon productivity? Are the effects of ETS on carbon productivity in different regions and industries consistent? To solve these problems, we explore how China’s pilot ETS affects regional industrial-level carbon productivity using a difference-in-difference-in-difference (DDD) model. Furthermore, we investigate the heterogeneity of ETS effects from regional and industrial perspectives and investigate the mediators.

This article contributes in the following aspects. Firstly, it empirically evaluates the impact of the pilot ETS on carbon productivity, which supplements relevant studies on carbon trading. Since only some high energy-consuming industries are covered by China’s pilot ETS, we employ the provincial industrial-level data that is in line with ETS’s coverage to obtain more reasonable and accurate estimates. Secondly, to avoid interference from other policies, we apply the cutting-edge DDD model to evaluate ETS’s effect. On this basis, we further explore the effect heterogeneity on different pilot regions and industries, providing a basis for formulating differentiated implementation plans for the ETS. Thirdly, the paper studies the mediating effect of ETS on carbon productivity, which can provide policy references of better operation for national ETS, and provide experience of carbon markets for other developing countries.

## 2. Literature Review

Current research on ETS has generally focused on two areas. The first is to study how to enhance the effectiveness of ETS. The second is to explore ETS’s impacts on social-economic factors. For the first area, most existing studies have discussed carbon prices. Some have found that the setting of carbon prices is a key factor for the ETS to effectively achieve emission reductions [4,21]. The ETS would be enhanced substantially if the carbon price and the carbon trading scale can be accurately predicted [22,23,24]. Others believe that the initial emission allowances allocation matters most for the effective operation of ETS [25].

In terms of the impacts of ETS, related studies have formed two groups from the perspective of research approaches. The first is simulation research based on the computable general equilibrium (CGE) model [26,27] or numerical simulation [28]. Those studies analyzed the potential impact of ETS on the environment or economy, but they may not fully reflect the real effect of ETS since the simulation is heavily influenced by parametric assumptions [29]. The other is regression analysis based on actual data. Some scholars found that the EU ETS was not fully effective in carbon emission reduction in power markets [30]. A series of recent studies confirmed that China’s pilot ETS substantially reduced carbon emissions in covered regions [17,31,32]. Additionally, some argued that China’s ETS also promoted carbon intensity reduction [33], but Zhang et al. (2019) [34] found that only in some pilot areas (e.g., Beijing and Guangdong) ETS decreased carbon emission intensity. Existing studies also stated that the implementation of China’s ETS can reduce energy consumption and intensity [31], boost new energy use [35], and influence technological innovation [36,37] and green development efficiency [38].

Overall, these studies mainly focus either on carbon emissions of a whole region (e.g., a province) [17,34,38] or all industries [33], which is inconsistent with the reality of pilot ETS coverage. Although Zhang et al. (2019) [31] and Hu et al. (2020) [32] tried to revolve around the specific covered industries, they use the difference-in-difference (DID) or propensity score matching (PSM) difference-in-difference (PSM-DID) methods which cannot eliminate the interference effects of other policies (e.g., national or regional industrial policies) [39,40], thereby reducing the validity of the estimated results.

Carbon productivity is the level of gross output (or economic value output) per unit of carbon emissions [41,42]. It measures the comprehensive level of low-carbon technologies over a certain period of time [43]. Increasing carbon productivity is an important way to achieve low-carbon transformation of economic modes [44]. It is estimated that China needs to increase its carbon productivity by a factor of 10 times to achieve the greenhouse gas emission target of IPCC2025 [45]. The existing literature has found carbon productivity is affected by economic scale [46], green capital investment [47], technological innovation level [48], trade openness degree [12,49], energy consumption structure [47], and urbanization level [13]. However, few have analyzed the effect of ETS on carbon productivity and the associated mediators.

In summary, the existing literature still has some shortcomings. Firstly, few have empirically studied the impact and mechanism of China’s ETS on carbon productivity. Secondly, existing studies have mostly used provincial-level data for the whole region or industrial-level data for all industries, which overestimated the coverage of China’s pilot ETS, and the estimated policy effects may not be accurate. Thirdly, most studies use DID or PSM-DID models, which cannot avoid the impact of other policies on carbon emissions. Therefore, to fill these gaps, we empirically assess the impact of China’s pilot ETS on carbon productivity using the DDD model and the regional industrial-level data reflecting the actual coverage of the pilot ETS.

## 3. Methods and Data

### 3.1. Methods

#### 3.1.1. Difference-in-Difference-in-Difference (DDD) Model

The difference-in-difference (DID) model is a widely applied method for policy effectiveness evaluation. However, the effects of many confounding factors, such as regional heterogeneity and other regulation policies, cannot be eliminated by DID, which may result in inaccurate results [39,40]. Therefore, we employ the DDD model [16,50] to tackle the above problems. Specifically, this paper regards the pilot industrial sectors in the pilot and non-pilot areas as the first group of the treatment group and control group, and regards the non-pilot industrial sectors in the pilot and non-pilot areas as another. Since the non-pilot industrial sectors are not affected by the pilot ETS, such a difference can eliminate other confounding factors, and further divest the net effectiveness of the pilot ETS [51]. The DDD model used is as follows:

(1) $$\ln Y_{i}{{}_{j}}_{t}=\beta_{0}+\beta_{1}time\times treat\times group+\lambda X+\gamma_{it}+\eta_{t}{}_{j}+\varepsilon_{i}{{}_{j}}_{t}$$

where, $Y_{ijt}$ is the dependent variable, indicating the carbon productivity of industry *j* located in region *i* in year *t*. It is specified in logarithmic form to observe the relative change. The term time denotes a year dummy variable, equaling 1 after the establishment of the pilot ETS (2013) or 0 otherwise. The term treat denotes a location dummy variable, equaling 1 if the region has its own CTM or 0 otherwise. The term group denotes another dummy variable and equals 1 if industry *j* is covered by the pilot ETS, otherwise the value is 0. The term X is a set of control variables, $\gamma_{i}{}_{t}$ denotes the province–year fixed effect, and $\eta_{t}{}_{j}$ denotes industry-year fixed effect. The term $\beta_{1}$ indicates the degree that the pilot ETS influences the carbon productivity of covered industries relative to the uncovered ones, and $\varepsilon_{i}{{}_{j}}_{t}$ is the random error term.

#### 3.1.2. Regional DDD Model

To figure out the regional heterogeneity of the pilot ETS’s influence, this paper selects all the pilot regions as samples and introduces the region dummy variable province. When a certain pilot region is investigated, the value of this area is 1, otherwise it is 0. It is combined with time×group to construct a triple interaction item to observe the difference in policy effectiveness between one pilot region and the others. The model is as presented in model (2). Other variables are the same as those in the model (1). The term $\beta_{2}$ of varying pilot regions can be used to derive the different policy effects between differing pilot regions.

(2) $$\ln Y_{i}{{}_{j}}_{t}=\beta_{0}+\beta_{2}time\times group\times province+\lambda_{1}X+\gamma_{i}{}_{t}+\eta_{t}{}_{j}+\varepsilon_{i}{{}_{j}}_{t}$$

#### 3.1.3. Industrial DDD Model

To verify the heterogeneity of policy effectiveness between industries, this paper introduces the industry dummy variable industry. When a certain industry covered by pilot ETS is investigated, the value is 1, otherwise it is 0. The term $\beta_{3}$ of different pilot industrial sectors can be used to derive varying policy effects between various pilot industries. Other variables are the same as those in model (1). The industrial heterogeneity DDD model is as follows:

(3) $$\ln Y_{i}{{}_{j}}_{t}=\beta_{0}+\beta_{3}time\times treat\times industry+\lambda_{1}X+\gamma_{i}{}_{t}+\eta_{t}{}_{j}+\varepsilon_{i}{{}_{j}}_{t}$$

#### 3.1.4. Stepwise Method

We adopt the mediating effect method [52,53] to explore how the pilot ETS influences carbon productivity. The model is as follows.

(4) $$\ln Y_{i}{{}_{j}}_{t}=\alpha_{1}time\times treat\times group+\lambda_{a}X+\gamma_{it}+\eta_{t}{}_{j}+\varepsilon_{i}{{}_{j}}_{t}$$

(5) $$M_{ijt}=\alpha_{2}time\times treat\times group+\lambda_{b}X+\gamma_{it}+\eta_{tj}+\varepsilon_{ijt}$$

(6) $$\ln Y_{ijt}=\alpha_{3}time\times treat\times group+\alpha_{4}M_{ijt}+\lambda_{c}X+\gamma_{it}+\eta_{tj}+\varepsilon_{ijt}$$

where, $M_{ijt}$ denotes mediators. Other variables are the same as in model (1). If $\alpha_{1}$, $\alpha_{2}$ and $\alpha_{4}$ are all significant, the mediating effect exists. Then $\alpha_{3}$ is further investigated. An insignificant $\alpha_{3}$ implies a complete mediating effect. If $\alpha_{3}$ is significant and its absolute value is less than $\alpha_{1}$, the mediating effect is partial-mediating.

### 3.2. Data

The data used include 34 industries (details are shown in Table A1 of the Appendix A) in the provinces of China’s mainland from 2008 to 2017. Shanghai and Tibet are not included because of data unavailability. In 2013, Beijing, Tianjin, Shanghai, Hubei, Chongqing, Guangdong and Shenzhen officially started their CTMs. Shanghai’s data is incomplete, and Shenzhen’s data have been statistically included in the data of Guangdong. Thus, Beijing, Guangdong, Hubei, Chongqing and Tianjin are finally selected as pilot regions. We summarize industries in the light of Industrial Classification for National Economic Activities [31]. Eight industries covered by the ETS in pilot regions are set as pilot industries. Details are shown in Table A2 of the Appendix A.

#### 3.2.1. Dependent Variable

Carbon productivity is calculated as follows [43]:

(7) $$CP_{j}^{it}=\frac{GOP_{j}^{it}}{CE_{j}^{it}}$$

where $CP_{j}^{it}$ denotes the carbon productivity of industry *j* located in area *i* in year *t* and $GOP_{j}^{it}$ denotes the industrial gross output, collected from the China Industry Statistical Yearbook [54]. It has been adjusted to a 2008 constant price by using the Industrial Producer Price Index [55]. $CE_{j}^{it}$ denotes the industrial carbon emissions, accounted for by the IPCC accounting method [56] as Equation (8).

(8) $$CE_{j}{}^{it}=\sum_{k}AD_{jk}^{it}\times NCV_{k}\times CC_{k}\times O_{k}\times \frac{44}{12}$$

where, $AD_{jk}^{it}$ denotes the consumption of energy *k* of industry *j* in region *i* in year *t*. The term $NCV_{k}$ denotes the mean low calorific value of energy *k*, $CC_{k}$ denotes carbon content of energy *k*, $O_{k}$ denotes the carbon oxidation rate of energy, and $\frac{44}{12}$ denotes the molecular weight ratio. Energy consumption data are collected from the China Energy Statistical Yearbook [57]. The terms $NCV_{k}$, $CC_{k}$ and $O_{k}$ are from IPCC [56,58]. Coal, crude oil, and natural gas are selected as energy sources for carbon emissions accounting because they account for about 94% of China’s total energy consumption [43]. We only consider direct emissions, excluding indirect emissions and process emissions in the measurement.

#### 3.2.2. Control Variables

The scales of industries have impact on productivity and profit margins and can affect the investments in energy-efficient equipment as well as technological innovations [59]. Accordingly, they may further influence industrial sectors’ carbon productivity. Therefore, we control the industrial scale with total assets and industrial average number of employees in the analysis [60,61]. The data are from the China Industry Statistical Yearbook [54]. Total assets are measured with a 2008 constant price utilizing the fixed asset investment price index [55].

The asset–liability ratio is an indicator of long-term solvency, which could affect the willingness of environmental management [62]. The asset profit ratio is an indicator of profitability, which may affect energy efficiency through capital investment [63] and technological innovation [64,65]. Moreover, the degree of asset liquidity is a main influential factor of technological innovation effectiveness [66]. Thus, we also control the asset–liability ratio, the asset profit ratio and the current assets ratio in the empirical analysis. These data are from the China Industry Statistical Yearbook (Department of Industry Statistics 2008–2018). Table 2 shows the interpretation and calculation of the control variables.

#### 3.2.3. Mediators

Technological progress has been widely measured by total factor productivity (TFP) [67,68]. Levinsohn and Petrin (2003) [69] proposed a semi-parametric method to measure TFP (LP method for short). The LP method can solve the problem of sample loss caused by a sample with zero investment that cannot be estimated by replacing variables. Therefore, we choose the LP method to measure TFP. The indicators used to calculate TFP have been adjusted to a 2008 constant price.

Capital intensity is selected to measure capital investment [19]. It is calculated as the proportion of annual capital investment over total output value for each industry.

## 4. Results and Discussions

### 4.1. The Overall Impact of ETS

We use the DDD model (Equation (1)) for measuring the overall effect of the pilot ETS on carbon productivity. Column (1) of Table 3 provides the estimates only controlled by the industry-year fixed effect, without control variables. The interaction term coefficient is significantly positive at the 1% level. Column (2) further controls the province–year fixed effect upon Column (1). The interaction term coefficient is still significantly positive, but it decreases from 1.2682 to 0.5631. Thus, it can be seen that the confounding factors varying with time in different regions are indeed interfering with the results. The R-squared value has improved, which shows that the model fits better after controlling time-varying factors between regions. Column (3) considers control variables and the industry-year fixed effect. The interaction term coefficient is also significantly positive, and its absolute value is smaller than that of Column (1), verifying that those industrial characteristics had a certain impact on carbon productivity. In Column (4), control variables and industry-year fixed and province–year fixed effects are all considered. The interaction term coefficient is significantly positive, and the carbon productivity of the treatment group increased by 58.25% after the pilot ETS implementation, compared to the control group. This proves that the pilot ETS can significantly increase the carbon productivity.

What does the effect of ETS look like in other countries? The European Union carbon market (EUCM) was first established globally. However, due to the over-lax quota allocation in the first phase of the EUCM (2005–2007) and free allocation as well as the overall economic downturn in the second phase (2008–2012), the effect of the EUCM was not effectively achieved [70]. Accordingly, EUCM began to reduce the total amount of quotas in the third stage (2013–2020), and the quota allocation was gradually shifted from free distribution to auction [70]. Nevertheless, EUCM cannot produce effective innovation incentives at all stages [71,72]. The second national-level ETS is the New Zealand ETS (NZ ETS), which is the most comprehensive one, covering all sectors and greenhouse gases. Overall, the NZ ETS has slowly improved, while remaining relatively dynamic, but the market has not increased emission reductions because the transactions are for purchasing eligible offset units [73]. With the CEG model, Meng et al. (2018) [74] found that Australia’s ETS can effectively reduce CO_2_ emissions, but Australia’s ETS emission reductions have not reached their target yet, and have caused the economy’s contraction as well as a reduction of the employment level [75]. South Korea launched an ETS (KETS) in 2015, but the incentives were insufficient as the carbon price was lower than the marginal abatement cost. As a result, KETS did not have a positive impact on the efficiency of coal-fueled power plants, the main participants of KETS, in 2015–2016 [76]. In 2020, the carbon price of KETS is the highest among all the carbon markets [77]. In general, worldwide ETSs still need to be further improved. Among them, China’s pilot ETS is one of the most effective carbon markets in terms of emissions reduction.

### 4.2. Heterogeneity Analysis Results

#### 4.2.1. Regional Heterogeneity

China’s pilot CTMs are implemented by local governments without a unified set of standards, so the effectiveness of the ETS may be different. We analyze the heterogeneity of ETS effectiveness in pilot regions.

Table 4 addresses the condition that only the interaction terms coefficients for Beijing and Chongqing are significant, indicating that the effectiveness of the pilot ETS is regionally heterogeneous. Beijing has the only significantly positive coefficient, indicating that Beijing’s pilot CTM had promoted carbon productivity increase.

There may be some potential explanations. First, the average unit carbon price in Beijing is much higher than that of the other pilot regions (Table 1). Higher carbon price is an effective incentive for enterprises to adopt emission reduction measures that are conducive to carbon productivity increase [32]. Second, more enterprises are covered by the pilot CTM in Beijing [78], boosting more active transactions and promoting the ETS’s scale effect. Third, the pilot ETS implementation is sturdy and the quality control of emissions data evaluation is strict in Beijing. Necessary guidelines and documents have been proposed to implement quality management from both the verification agencies and the inspectors. In the meanwhile, a team of experts has also been established for checking all the verification reports. Finally, it may also be related to Beijing’s political status. As the capital and political center of China, the implementation of the pilot ETS is more likely to be run with tireless efforts.

Chongqing’s coefficient is significantly negative, implying a less effective CTM. The result is in accordance with Zhang et al. (2019b) who found that the CTM had limited effect on Chongqing’s carbon emission intensity. Relatively low carbon price may be one of the reasons (Table 1). This may also be affected by the way carbon quotas are allocated. Beijing and Tianjin adopt the “historical intensity method” and Guangdong adopts the “baseline method”. The method in Hubei is a combination of the two. For Chongqing, a self-declaration method is employed, resulting in sufficient and even oversupply of the carbon quota. In that case, the carbon market may not enhance carbon productivity substantially.

#### 4.2.2. Industrial Heterogeneity

We also explore the industrial heterogeneity effect of the pilot ETS. Petrochemical and electric power industries have significantly positive interaction term coefficients, and petrochemical industry has the largest absolute value (1.3908) (Table 4). This indicates that the pilot ETS strongly promoted the carbon productivity of those two industries. In contrast, the coefficients of building materials (−0.5102) and transportation (−0.9184) are significantly negative.

These results may be affected by the differences in industrial emission reduction potential. Wang et al. (2017) reported that the petrochemical industry has the largest emission reduction potential, followed by the electric power industry, and the transportation industry has the smallest one. The emission reduction potential of the building materials industry is also relatively small. In the petrochemical industry, carbon productivity can be greatly improved by expanding the proportion of clean energies used and using emission-reducing technologies. The power industry can also greatly promote carbon productivity through large-scale onshore wind power generation and high-efficiency natural gas power generation technologies.

Due to the heterogeneity among industries, the allocation of carbon quotas should also be differentiated. Setting large emission reduction pressures on industries with small emission reduction potential will affect their economic output, and some foreign trade industries may even have carbon leakage [79]. It is more appropriate to consider the industrial emission reduction potential in carbon quota allocation to maximize the overall improvement of carbon productivity.

### 4.3. Mediating Effects Results

#### 4.3.1. Technological Progress

Empirical evidence supports the position that in the face of carbon emission quota constraints, enterprises will reduce emissions through technological progress to achieve low-carbon production [80]. Therefore, we further consider the potential mediating effect of technological progress. Column (1) of Table 5 reports the overall effect, which is the regression result of Equation (3). The positive interaction term coefficient indicates that the pilot ETS promoted carbon productivity. The dependent variables in Columns (2) and (3) are the mediator *Lntfp*. Column (3) adds the province–year fixed effect on the basis of (2), absorbing the provincial time-varying factors. The coefficient of *time × treat × group* is also significantly positive, indicating that the pilot ETS promoted technological progress effectively. Column (4) reports the result of Equation (5), which includes *time × treat × group* and *Lntfp* simultaneously. The coefficients of interaction term and *Lntfp* are all significant, and the absolute value of the interaction term coefficient is smaller than that of Equation (1). So far, it can be proved that the mediating effect of technological progress exists, which is partial-mediating. This shows that the pilot ETS increased carbon productivity through technological progress.

We provide the potential explanation. The ETS motivates enterprises to adopt emission reduction measures through cost pressures caused by carbon quota constraints [81] and benefit incentives brought by the marketization mechanism of policies [82]. For the long-term, enterprises are likely to choose the measures that are more conducive to their long-term development, such as technological progress, to achieve low-carbon production [83]. Technological progress includes technological innovation and improvement. The impact of the former is mainly achieved by low-carbon R&D. The latter is through upgrading equipment, improving processing conversion efficiency, using waste heat and pressure resources for cyclic production, and optimizing the allocation of production resources [84].

#### 4.3.2. Capital Investment

The results of capital investment as a mediator are shown in Table 5. Column (5) reports the overall effect, with *Lncp* as the dependent variable. The dependent variable is capital intensity (*CI*) in both Columns (6) and (7). Column (7) adds the provincial fixed effect on the basis of Column (6), absorbing mutual unobservable factors at the regional level. The interaction term coefficient implies that the pilot ETS significantly increased capital investment, notwithstanding that the coefficient’s absolute value is small, indicating that the pilot ETS played a smaller role in increasing capital investment than in promoting technological progress. Column (8) includes capital investment (*CI*) in the overall effect regression model. The coefficients of *time × treat × group* and *CI* are both significantly positive, and the absolute value of the *time × treat × group* coefficient is smaller than the counterpart in Column (1). So far, the mediating effect of capital investment can be proved, which is partial-mediating. Nevertheless, the interaction term coefficient in Column (8) is only slightly smaller than that in Column (5). It can be seen that the effect of capital investment was weaker than that of technological progress on carbon productivity improvement. This shows that enterprises preferred to promote technological progress rather than increase capital investment. A potential reason is that capital investment such as fixed assets investment may incur pressure on capital flow, but technological progress can be achieved by internal resources optimization, so enterprises may be more inclined to choose the latter.

### 4.4. Robustness Test Results

#### 4.4.1. Placebo Test

**Figure 1:**
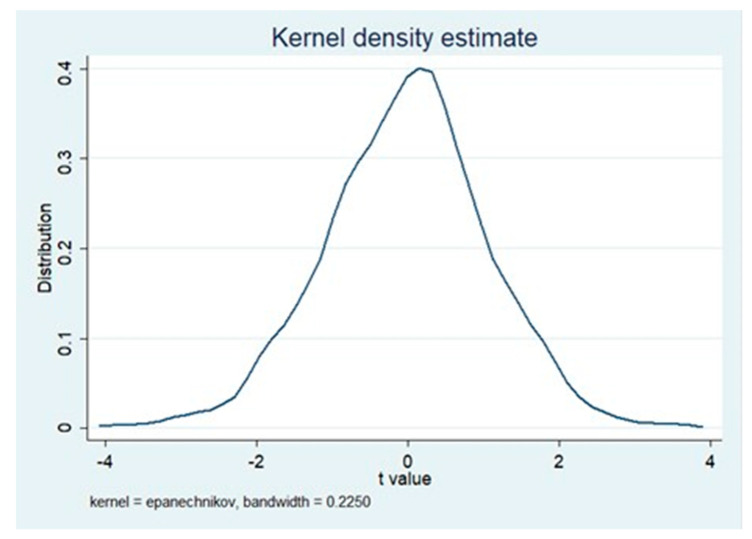
Placebo test results.

We adopted a placebo test [85] to identify whether our results are driven by unobservable factors at regional, industrial, and year levels. Specifically, a treatment group of the pilot ETS was randomly set to ensure that the selection of the pilot did not affect the dependent variable, that is, the interaction term coefficient of random regression equals 0. We conducted 1000 random samplings and performed regression according to Formula (1). Figure 1 reports the *t*-value distribution of 1000 results, most of which are distributed near zero. The mean of the coefficient after random sampling is 0.0017, which is close to 0 compared to the above DDD model results and is not significant. This proves that our estimates are hardly affected by unobservable factors at the regional, industrial and year levels.

#### 4.4.2. Concurrent Event Test

During 2013–2014, other laws and regulations on carbon emissions or energy use issued by China may affect our results. We therefore consider the policy event as it may bias the conclusion. In 2014, China began to implement pilot water rights trading in six provinces, including Hubei. The water rights trading mainly deals with regional water use for industrial water transactions [86]. Water consumption is an important factor affecting industrial production. The implementation of this policy may have an impact on the output of the industries, which in turn may reduce energy consumption and carbon emissions. Therefore, a robustness test was performed in this paper. Following Shi and Xu (2018), Ningxia, Jiangxi, Hubei, Inner Mongolia, Henan, and Gansu provinces with pilot water rights trading are eliminated, and regression testing is performed using model (1). The significant triple interaction term implies that carbon productivity improvement has not been affected by the pilot water rights trading (Table 6).

## 5. Conclusions

In this paper, China’s pilot ETS is regarded as a quasi-natural experiment. Using provincial industrial-level data, the effect of the pilot ETS on carbon productivity is explored by DDD model. In addition, the regional and industrial heterogeneous effects and the mediating effects of technical progress and capital investment are also analyzed. We find that the pilot ETS increased carbon productivity of the coverage of China’s pilot ETS. Among pilot regions, the best policy effectiveness appeared in Beijing while the weakest effectiveness appeared in Chongqing. Among the pilot industries, and the pilot ETS had better effectiveness in petrochemical and electric power industries and weaker effectiveness in building materials and transportation industries. Moreover, the pilot ETS improved carbon productivity by promoting technological progress and capital investment and the former contributed more.

Several policy implications are proposed. First, it is of great importance to set up appropriate and reasonable carbon quota allocation methods. More appropriate carbon quota allocations need to be formulated according to the conditions of each region. Second, different emission reduction pressures can be set according to the industry’s emission reduction potential. Industries with large potentials can more reasonably and efficiently increase carbon productivity and promote a low-carbon economy. Third, it is necessary to establish a dynamic carbon quota adjustment program. For regions with low carbon prices, the authorities can appropriately tighten carbon quotas to stimulate the price of carbon emissions trading.

Further research can combine enterprise-level data to comprehensively analyze the impact mechanism of China’s pilot ETS on carbon productivity, and come up with more targeted policy recommendations from the enterprise perspective. Besides, the policy spillover effect of China’s pilot ETS also needs further study.

## Figures and Tables

**Table 1:** Pilot emissions trading scheme in China.

Bourse	Brand	Time	Turnover (10^6^ ¥)	Trading Volume (10^5^ ton)	Average Unit Transaction Price (¥/ton)
Beijing	BEA	28 November 2013–31 December 2017	358.86	71.20	50.40
Guangdong	GDEA	19 December 2013–31 December 2017	558.02	384.85	14.50
Tianjin	TJEA	26 December 2013–31 December 2017	41.16	30.05	13.70
Hubei	HBEA	2 April 2014–31 December 2017	911.10	489.16	18.63
Chongqing	CQEA	19 June 2014–31 December 2017	30.05	75.13	4.00

Note: 1 US dollar ($) = 6.9 Chinese yuan (**¥**). Turnover refers to the total transaction value of each bourse from initial operating time to 31 December 2017.

**Table 2:** Summary statistics.

Variable Type	Variable	Symbol	Variable Meaning	Mean	Standard Deviation
Dependent variable	Carbon productivity	*Lncp*	Industrial carbon emissions/industrial total output value (in log) (10^6^t/10^8^ RMB)	1.6376	2.2057
Control variable	Industrial scale	*Lnasset*	Industrial total assets in log (10^8^ RMB)	4.9014	2.0799
		*Lnlabor*	Industrial average number of employees in log (10^4^ people)	1.4347	1.0586
	Asset-liability ratio	*AL*	Industrial total liabilities/industrial total assets × 100%	83.3002	344.2183
	Asset profit ratio	*AP*	Industrial total profit/industrial total assets × 100%	10.4222	43.9045
	Current assets ratio	*CA*	Industrial total current assets/industrial total assets × 100%	45.1310	19.0907
Mediator	Technological progress	*Lntfp*	Total factor productivity	1.9317	2.3115
	Capital investment	*CI*	Industrial fixed assets investment/industrial total output value	19.8296	984.4881

Note: 1 US dollar = 6.9 Chinese yuan (RMB).

**Table 3:** Impact of ETS on carbon productivity.

Variables	(1)	(2)	(3)	(4)
*ttg*	1.2682 *** (0.2526)	0.5631 ** (0.2824)	1.1626 *** (0.2665)	0.5825 ** (0.2803)
*_cons*	1.6070 *** (0.6739)	1.6240 *** (0.0068)	0.1491 (0.2487)	0.8154 *** (0.2749)
*Controls*	N	N	Y	Y
*Iyfe*	Y	Y	Y	Y
*Pyfe*	N	Y	N	Y
R-squared	0.4570	0.6149	0.4950	0.6301

Note: Standard errors are clustered at the industrial units; ** and *** indicate significance at the 5% and 1% levels, respectively. *ttg* denotes *time × treat × group. Iyfe* and *Pyfe* denote industry–year and province–year fixed effects, respectively. Y denotes that the variables are added and N denotes that the variables are not included.

**Table 4:** Heterogeneity analysis results.

Variables	Regional Heterogeneity					Industrial Heterogeneity							
	Beijing	Guangdong	Tianjin	Hubei	Chongqing	Papermaking	Petrochemical	Chemical	Building Materials	Steel	Non-Ferrous Metal	Transportation	Electric Power
*ttg*	1.0721 *** (0.3691)	0.7366 (0.4581)	0.0196 (0.6093)	−0.4756 (0.5124)	−1.2894 ** (0.5349)	−0.1101 (0.2862)	1.3908 *** (0.1883)	0.0443 (0.2605)	−0.5102 * (0.2524)	−0.3486 (0.3015)	−0.3420 (0.2590)	−0.9184 ** (0.2756)	0.7409 ** (0.3037)
*_cons*	1.0624 (0.9379)	1.1469 (0.9205)	1.2036 (0.9590)	1.2030 (0.9502)	1.1436 (0.8987)	0.4128 (0.4678)	0.3108 (0.5124)	0.4132 (0.4611)	0.4166 (0.4657)	0.4181 (0.4634)	0.4482 (0.4640)	0.3404 (0.4860)	0.4334 (0.4490)
*Controls*	Y	Y	Y	Y	Y	Y	Y	Y	Y	Y	Y	Y	Y
*Iyfe*	Y	Y	Y	Y	Y	Y	Y	Y	Y	Y	Y	Y	Y
*Pyfe*	Y	Y	Y	Y	Y	Y	Y	Y	Y	Y	Y	Y	Y
R-squared	0.6673	0.6650	0.6628	0.6638	0.6697	0.6996	0.7041	0.6996	0.7002	0.6999	0.6999	0.7014	0.7009

Note: Standard errors are clustered at the industrial units; *, ** and *** indicate significance at the 10%, 5% and 1% levels, respectively. *ttg* denotes *time* × *treat* × *group*. *Iyfe* denotes industry-year fixed effect. *Pyfe* denotes province–year fixed effect. Y denotes that the variables are added and N denotes that the variables are not included.

**Table 5:** Mediating effect results.

Variables	Technological Progress				Capital Investment			
	(1)	(2)	(3)	(4)	(5)	(6)	(7)	(8)
*ttg*	0.5825 ** (0.2803)			0.4845 * (0.2801)	0.5825 ** (0.2803)			0.5809 ** (0.2805)
*Lntfp*		0.6303 *** (0.1303)	0.2125 * (0.1168)	0.2228 *** (0.0453)				
*CI*						0.0549 * (0.0285)	0.0682 * (0.1168)	0.0098 *** (0.0453)
*_cons*	0.8154 *** (0.2749)	−1.2764 *** (0.2730)	−1.0281 *** (0.2482)	1.3141 *** (0.2773)	0.8154 *** (0.2749)	1.0645 *** (0.3611)	−1.6171 (1.8374)	1.3141 *** (0.0026)
*Controls*	Y	Y	Y	Y	Y	Y	Y	Y
*Iyfe*	Y	Y	Y	Y	Y	Y	Y	Y
*Pyfe*	Y	N	Y	Y	Y	N	N	Y
*Pfe*	N	N	N	N	N	N	Y	N
R-squared	0.6301	0.2218	0.8348	0.6400	0.6301	0.0877	0.0883	0.6304

Note: Standard errors are clustered at the industrial units; *, ** and *** indicate significance at the 10%, 5% and 1% levels, respectively. The term *ttg* denotes *time × treat × group*, *Iyfe* denotes the industry–year fixed effect, *Pyfe* denotes the province–year fixed effect, *Pfe* denotes the province fixed effect, Y denotes that the variables are added, and N denotes that the variables are not included.

**Table 6:** Concurrent event inspection.

Variables	Pilot Water Rights Trading	
	(1)	(2)
*time × treat × group*	1.1323 ***	0.5355 **
	(0.1763)	(0.2353)
*_cons*	−0.0560 *	0.5528 **
	(0.2999)	(0.3102)
*Controls*	Y	Y
*Pyfe*	N	Y
*Iyfet*	Y	Y
*R-squared*	0.4668	0.6125

Note: Standard errors are clustered at the industrial units; *, ** and *** indicate significance at the 10%, 5% and 1% levels, respectively, *Iyfe* denotes the industry–year fixed effect, *Pyfe* denotes the province–year fixed effect, Y denotes that the variables are added and N denotes that the variables are not included.

## Abbreviations

ETS	emissions trading scheme
CTM	carbon trading market
DDD	difference-in-difference-in-difference
DID	difference-in-difference
PSM-DID	propensity score matching difference-in-difference

## Appendix A

**Table A1:** List of sample industries.

Industry Classification Name	Code	Industry Classification Name	Code
Mining and Washing of Coal	B06	Processing of Petroleum, Coking, Processing of Nuclear Fuel	C25
Extraction of Petroleum and Natural Gas	B07	Manufacture of Raw Chemical Materials and Chemical Products.	C26
Mining and Processing of Ferrous Metal Ores	B08	Manufacture of Medicines	C27
Mining and Processing of Non-Ferrous Metal Ores	B09	Manufacture of Chemical Fibers	C28
Mining and Processing of Nonmetal Ores	B10	Manufacture of Rubber and Plastics	C29
Processing of Food from Agriculture Products	C13	Manufacture of Non-Metallic Mineral Products	C30
Manufacture of Foods	C14	Smelting and Pressing of Ferrous Metals	C31
Manufacture of Beverages	C15	Smelting and Pressing of Non-Ferrous Metals	C32
Manufacture of Tobacco	C16	Manufacture of Metal Products	C33
Manufacture of Textile	C17	Manufacture of General Purpose Machinery	C34
Manufacture of Textile Wearing Apparel, Footware and Caps	C18	Manufacture of Special Purpose Machinery	C35
Manufacture of Leather, Furs, Feather and Related Products	C19	Manufacture of Transport Equipment	C36, C37
Processing of Timber, Manufacture of Wood, Bamboo, Rattan, Palmand Straw Products	C20	Manufacture of Electrical Machinery and Equipment	C38
Manufacture of Furniture	C21	Manufacture of Communication Equipment, Computers and Other Electronic Equipment	C39
Manufacture of Paper and Paper Products	C22	Manufacture of Measuring Instruments and Machinery for Culture Activity and Office Work	C40
Printing, Reproduction of Recording Media	C23	Production and Distribution of Electric Power, Heat Power and Gas	D44, D45
Manufacture of Articles for Culture, Education and Sports Activity	C24	Production and Distribution of Water	D46

**Table A2:** List of pilot industries.

Pilot Industries	Industry Classification Name	Data Description
Papermaking	Papermaking and Paper Products	
Petrochemical	Petroleum Processing and Coking	
Chemical	Raw Chemical Materials and Chemical Products	
Building materials	Non-metal Mineral Products	
Steel	Smelting and Pressing of Ferrous Metals	
Non-ferrous metal	Smelting and Pressing of Nonferrous Metals	
Transportation	Transportation Equipment Manufacturing	Merge “Automotive Manufacturing” with “Railroad, Ships, Aerospace and Other Transportation Equipment Manufacturing”
Electric power	Production and Supply of Electric Power, Heat Power and Gas	Merge “Production and Supply of Electric Power and Heating Power” with “Production and Supply of Gas”
